# Ionizing Radiation Crosslinked Chitosan-Based Hydrogels for Environmental Remediation

**DOI:** 10.3390/gels11070492

**Published:** 2025-06-25

**Authors:** Muhammad Asim Raza

**Affiliations:** School of Chemical Engineering, Yeungnam University, Gyeongsan 38541, Republic of Korea; muhammadasimraza@yu.ac.kr

**Keywords:** chitosan, ionizing radiation, hydrogels, environmental remediation

## Abstract

Since water contamination has become a serious concern, more effective environmental remediation methods are required. Chitosan (CHT)-based adsorbents have demonstrated high efficacy in removing pollutants due to their unique chemical and structural properties. However, their utilization remains limited by low environmental stability and the absence of effective adsorption sites. The functional moieties of CHT can be altered to improve its performance via graft modification and crosslinking. Compared to conventional hydrogel synthesis techniques, ionizing radiation-induced fabrication, using gamma or electron-beam irradiation, offers a promising platform for innovation across diverse fields. The prime focus of this review is on ionizing radiation developed CHT-based hydrogels to remove toxic heavy metals, dyes, organic contaminants, radionuclides, and humic substances. The fabrication strategy, adsorption mechanism, and factors affecting the adsorption capacity of CHT-based hydrogels are presented. This review aims to underscore the transformative potential of ionizing radiation-induced CHT hydrogels in environmental remediation by examining current research trends and identifying future prospects.

## 1. Introduction

Hydrogels are polymeric materials characterized by their hydrophilic structure and ability to swell in water, enabling them to retain large quantities of water within their three-dimensional (3D) polymeric networks. This unique property has attracted increasing interest due to its relevance in various applications [1]. Natural polymer-based hydrogels have gained popularity owing to their biodegradability, environmental compatibility, abundant raw-material sources, and cost-effective production. Consequently, hydrogels have been synthesized through ionizing radiation-assisted polymerization using polymers such as chitosan (CHT), cellulose, and starch [2,3,4,5,6]. Radiation-induced polymerization presents several advantages over conventional methods, including one-step purification, straightforward process control, and the initiation of reactions without the need for additives or catalysts, regardless of temperature, pressure, or phase [7].

As industry and technology advance, non-renewable petrochemical resources are being depleted at an accelerating rate, contributing to increasingly severe global environmental pollution [8]. In this context, adsorption has emerged as one of the most effective, economical, and widely applied techniques for the removal of toxic heavy metal ions from contaminated aqueous solutions. The adsorption of metal ions can be achieved using macroradicals containing functional groups [9]. CHT, being one of the most abundant naturally occurring amino-polysaccharides, has garnered significant attention due to its unique biological activities and physicochemical properties [10,11]. The hydroxyl (–OH) and amino (–NH_2_) groups in CHT enable interactions with various pollutants. Specifically, the adsorption of heavy metal ions by CHT-based hydrogels is primarily facilitated through coordination and ion-exchange mechanisms [12]. The network structure of CHT-based hydrogels provides a high specific surface area and porosity, which enhances their adsorption capacity [13]. Moreover, these hydrogels exhibit stability across a wide range of pH levels and temperature variations [14,15], making them adaptable to complex natural environments. Their ability to tolerate fluctuations in soil and water conditions, including hardness, salinity, and other environmental factors, further supports their suitability for pollutant remediation.

This review systematically examines the current state of research on the fabrication of CHT-based hydrogels using ionizing radiation technology. It discusses the structure and properties of CHT, various fabrication methodologies, and multifunctional environmental remediation applications—including the removal of toxic heavy metals, dyes, organic contaminants, radionuclides, and humic substances. The review also evaluates the potential benefits and challenges associated with this approach.

## 2. Structure of Chitosan and Its Properties

Chitin is the most abundant natural polymer in nature after cellulose and is found in various eukaryotic organisms, including insects, crustaceans, and fungi [16,17]. CHT, obtained through the deacetylation of chitin, represents a structurally intriguing derivative of this biopolymer [18]. CHT is composed of repeating units of D-glucosamine and N-acetyl-D-glucosamine linked via β-(1→4) glycosidic bonds [19]. In this polysaccharide, the proportion of β-(1→4)-D-glucosamine units is defined as the degree of deacetylation (DD), while the mole fraction of N-acetylated repeating units corresponds to the degree of acetylation (DA) [20]. Typically, CHT exhibits DD values ranging from 40% to 75%, with most commercial variants possessing DD values between 70% and 90% [16,21]. Another fundamental parameter influencing the physicochemical and biological properties of CHT is its molecular weight (MW). Based on MW, CHT is categorized into oligosaccharides, and low, medium, or high molecular weight forms. Lower MW CHT, similar to DA, demonstrates enhanced bioactivity compared to its higher MW counterparts [22,23].

Uniquely, CHT is the only naturally occurring cationic polysaccharide, distinguished by its distinctive chemical structure that underpins a range of biological activities, including antioxidant activity, antimicrobial properties, biodegradability, and adhesive capabilities. Its antioxidant activity is primarily attributed to the presence of –NH_2_ and –OH groups integrated within its molecular framework. In particular, the –NH_2_ groups contribute by donating hydrogen atoms, released as ammonia, before forming macromolecular structures capable of neutralizing reactive free radicals [24].

**Figure 1:**
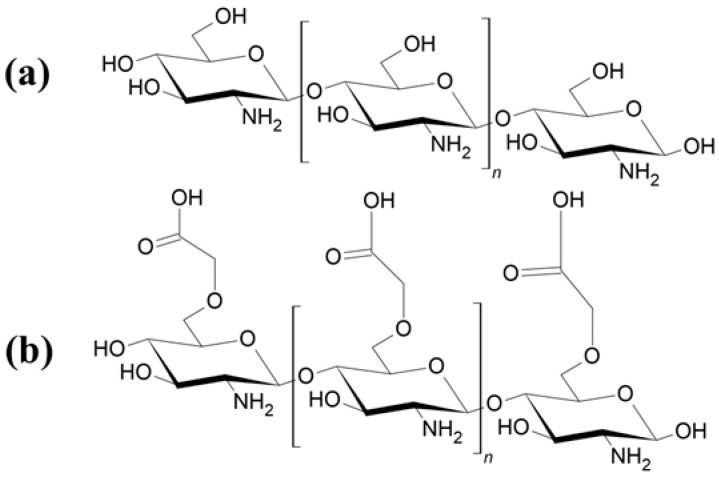
Chemical structures of (**a**) CHT and (**b**) CM-CHT.

However, a key limitation of CHT is its poor solubility at physiological pH levels (pH > 6), which complicates processing and limits its applicability. To overcome this constraint, various CHT derivatives—such as CHT esters and ethers—have been investigated [25,26,27]. One notable example is carboxymethyl CHT (CM-CHT), which incorporates carboxyl (–COOH) groups in addition to the native –OH and –NH_2_ functionalities. Figure 1 presents the chemical structures of CHT and CM-CHT. This modification enhances water solubility while preserving the high biocompatibility and antimicrobial properties characteristic of native CHT. Consequently, CM-CHT is well-suited for hydrogel applications in drug-delivery systems, biomedical fields, and adsorption technologies [28,29,30].

## 3. Crosslinking Strategies for Chitosan Hydrogel

The formation of hydrogel requires crosslinking, which generates a 3D-network structure and makes it insoluble in the medium [31]. Hydrogels are commonly crosslinked via three main methods: physical crosslinking, chemical crosslinking, and irradiation crosslinking. Physically crosslinked hydrogels generally exhibit low mechanical strength, whereas chemically crosslinked hydrogels often involve toxic reagents such as initiators or crosslinkers, which may raise environmental and biocompatibility concerns [32,33]. In contrast, ionizing radiation presents a simple and efficient alternative for hydrogel fabrication under ambient conditions, eliminating the need for additional chemical agents [34].

### 3.1. Physical Crosslinking

Physical crosslinking involves linkages of polymeric chains through physical interactions or non-chemical means, typically involving a combination of water and polymer components [35]. Physically crosslinked hydrogels generally exhibit low mechanical strength, a tendency to reverse the gelation process, and reduced toxicity. Physical crosslinking is further classified into ionic interaction, hydrogen-bonding interaction, hydrophobic interaction, and electrostatic interaction.

Ionic interaction is considered a well-known technique for creating physical crosslinking within polymers, effectually influencing and enhancing the crosslinking between polymeric chains. This develops a highly stable network structure, thus enhancing the stability and the mechanical strength of the hydrogel [36]. Hydrogen bonding is vital in the physical crosslinking of hydrogels, significantly impacting their characteristics, structure, and stability. Hydrogen-bonding interactions arise when a negatively charged acceptor atom, such as nitrogen, oxygen, or fluorine, forms an electrostatic connection with a positively charged hydrogen atom [37]. In hydrophobic interactions, hydrophobic molecules show attractive forces responsible for the properties and assembly of physically crosslinked hydrogels. These interactions are owing to cohesion-induced acid-base free energy between the water molecules. These forces facilitate the generation of larger aggregates of molecules in solutions instead of individual entities. Therefore, hydrophobic interactions fabricate physical crosslinked hydrogels by increasing the intermolecular attraction between polymeric units, and imbue the hydrogel with higher mechanical strength and stability [38]. Among physical crosslinking mechanisms, electrostatic interactions are most frequently used [39,40]. Electrostatic repulsion or attraction occurs between two charged objects when they are in the vicinity of each other and affects the stability of gel structure and molecular aggregation. Under pH conditions, the polyelectrolyte complexes are developed between cationic –NH_2_ groups in CHT and anionic groups in other polymers through these interactions.

### 3.2. Chemical Crosslinking

Chemical crosslinking generates hydrogels, which involves covalent linkages of –NH_2_ and –OH groups present on CHT chains with the crosslinking agent. These interactions result in a more stable and rigid hydrogel structure [41]. Hydrogels are generated by linking two polymeric molecules with a covalent bond in chemical crosslinking. These hydrogels are termed as true gels and provide a stable, irreversible, and permanent structure [42,43]. Chemical crosslinking is further classified into the Schiff base reaction, Michael addition reaction, thiol-ene reaction, Diels–Alder reaction, and nucleophilic ring opening reaction. Mostly the Schiff base reaction is carried out in acidic conditions to enable the condensation reaction between alcohol or ketone and the –NH_2_ group, to generate amide molecules [44]. The Michael addition reaction involves a conjugate addition reaction between an α, β-unsaturated compound having one or more carbon–carbon double bonds with attached functional groups and a nucleophilic reagent [45]. The thiol-ene reaction forms a carbon–sulfur single bond through the replacement of a hydrogen atom on the sulfur atom of a thiol group with a carbon atom on the carbon–carbon double bond of an olefin. Using this mechanism, novel bioactive and functional materials are generated by the modification of polymers [46]. The Diels–Alder reaction generates a six-member ring structure through the combination of a substituted alkene and a conjugated diene [47]. Medicinal compounds, natural products, and complex chemicals are synthesized using this reaction. Nucleophilic ring-opening reactions comprise the reaction between regions of high electron density in the reactants, and nucleophilic reagents, such as –NH_2_, alcohols, and thiols. The ring structure is disrupted and new chemical bonds are developed, which undergo the expansion of the ring structure or the generation of new compounds. This reaction involves the nucleophilic attack of ternary heteroatoms (such as nitrogen, oxygen, sulfur) within the molecule, subduing the cyclic strain and liberating the internal energy to develop ions like cyclic sulfates, cyclic sulfur ions, and epoxidized heterocyclic propane compounds [48].

### 3.3. Irradiation Crosslinking

**Figure 2:**
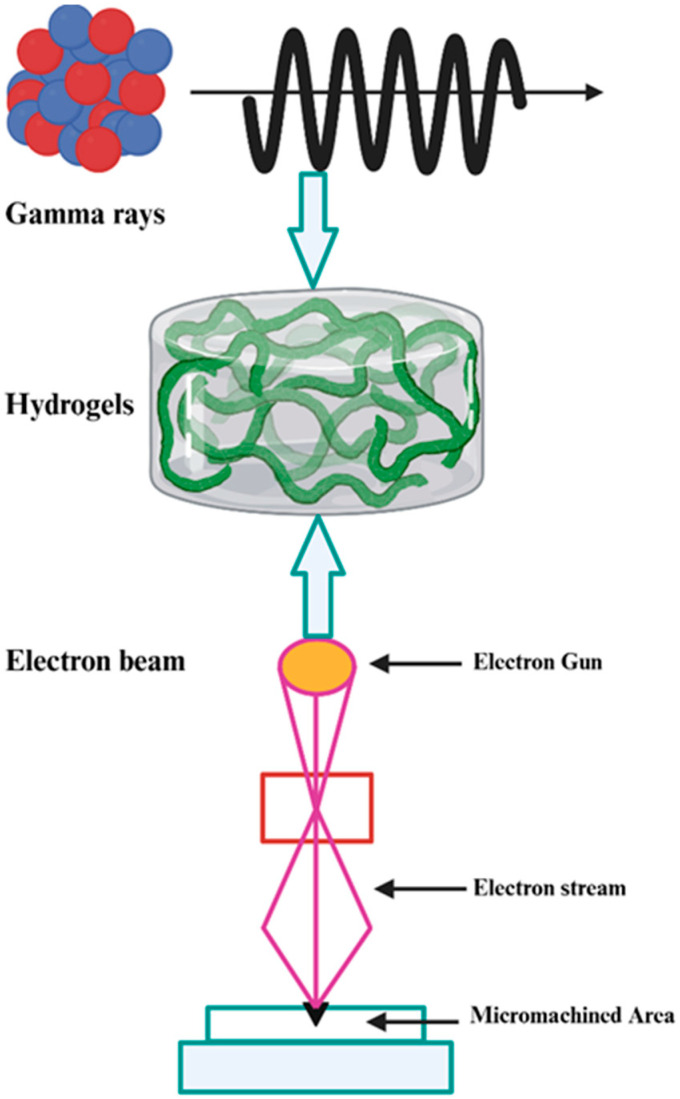
Schematic overview of hydrogel synthesis using ionizing radiation.

Radiation-based technologies offer several advantages, such as an environmentally friendly process for preparing cross-linked polymeric materials. A key benefit is that chemical reactions within the processed materials are initiated by radiation, eliminating the need for additional substances—often toxic—such as crosslinking agents, initiators, or other auxiliaries. This enables both the preparation and sterilization of materials in a single technological step, thereby reducing costs and simplifying the process. Furthermore, the approach minimizes the generation of waste and by-products, enhancing the overall environmental sustainability of the technology [49,50,51,52,53,54]. Within this context, gamma (γ) radiation and electron beam (E. beam) irradiation are the most frequently employed methods [55]. Ionizing radiation, such as γ irradiation, is considered a powerful tool for graft polymerization, as it does not require initiators or additives, and can be applied at any temperature in either a solution or solid phase. These properties are particularly advantageous for synthesizing biomaterials, where the absence of additional chemicals and the flexibility of processing conditions are critical [56,57]. γ-irradiation is regarded as one of the most effective and environmentally friendly crosslinking methods available [58]. The E. beam technique, in which high-energy electrons initiate radical reactions, also enables polymerization without the need for solvents, initiators, or other chemicals, while simultaneously sterilizing the material [59]. The energy of the electrons, typically in the keV or MeV range, determines their penetration depth in the sample [60]. Figure 2 illustrates the synthesis of hydrogels through ionizing radiation (γ-radiation and E. beam). There are certain limitations of this technology, which include the hefty purchase cost of a γ-irradiator or E. beam accelerator, and the maintenance costs are huge. Costs associated with acquiring a proper place, auxiliary equipment, control systems, process monitoring, suitable radiation shielding equipment, and the material-handling system are included in the initial investment. Besides this, authorization from regulatory bodies that monitor nuclear-related activities is also required.

## 4. Fabrication of Chitosan Hydrogels Using Ionizing Radiation

Several attempts have been made to modify the CHT structure through crosslinking and grafting techniques to enhance specific properties, such as the ability to absorb and retain large volumes of fluid. These modifications effectively merge the beneficial properties of synthetic and natural polymers [6,61,62,63,64]. Among these strategies, graft copolymerization of CHT with vinyl monomers, which introduces new functional groups, has become a preferred approach to improving its adsorption capacity [65]. This review discusses CHT-based systems incorporating natural polymers, synthetic polymers, and monomers. For instance, Wasikiewicz et al. irradiated highly concentrated (25–40%) CM-CHT and carboxymethyl chitin (CM-chitin) using an E. beam to produce hydrogels for metal ion adsorption [66]. The irradiation induced intermolecular crosslinking, resulting in the formation of insoluble gels with advantageous properties. Similarly, Hiroki et al. prepared high-concentration CM-CHT/carboxymethyl cellulose (CMC) blends and conducted crosslinking under paste-like conditions using γ-irradiation [67]. Elemental analysis confirmed that the composition of the resulting blend hydrogels corresponded to the initial CM-CHT/CMC ratios. As the radiation dose increased, the gel fraction increased while the swelling degree declined, indicating that both parameters could be tuned by adjusting the radiation dose and blend composition. In a separate study, Wang et al. grafted (2-methacryloyloxyethyl) trimethyl ammonium chloride (DMC), a quaternary ammonium salt, onto CHT using γ-irradiation [68]. The physicochemical properties of the resulting copolymer were evaluated, and the effects of various reaction parameters—including the irradiation dose rate, total absorbed dose, temperature, and monomer concentration—on the grafting percentage were systematically investigated. The results indicated that grafting efficiency increased with both temperature and total absorbed dose, up to 450 Gy. Additionally, higher monomer concentrations enhanced the availability of DMC molecules for reaction with CHT macroradicals, further increasing the grafting percentage. Moreover, Elbarbary et al. synthesized PAAM/CHT/sodium alginate (NaAlg) superabsorbent hydrogels via γ-radiation-induced crosslinking [69].

### Nanostructure Incorporated Chitosan-Based Nanocomposite Hydrogels

The integration of nanotechnology with biocompatible polymers has gained significant attention in recent years, with numerous efforts directed toward merging nanoscale techniques with conventional processes to enhance material performance. Nanocomposite hydrogels exemplify this convergence, combining the structural and functional advantages of nanostructures with the biocompatibility and versatility of polymeric hydrogels [70]. These systems exhibit unique physicochemical and biological properties that surpass those of the individual components [71]. In nanocomposites, nanostructures serve as reinforcing agents, while polymers function as the matrix, imparting thermal and chemical stability to the overall system [72]. This synergistic design expands the application potential of CHT-based hydrogels [73,74]. Incorporating nanomaterials such as graphene, silver nanoparticles (AgNPs), carbon nanotubes (CNTs), nanoclay, and magnetic nanoparticles into CHT matrices enhances not only their efficiency and adsorption capacity but also introduces novel functionalities.

**Figure 3:**
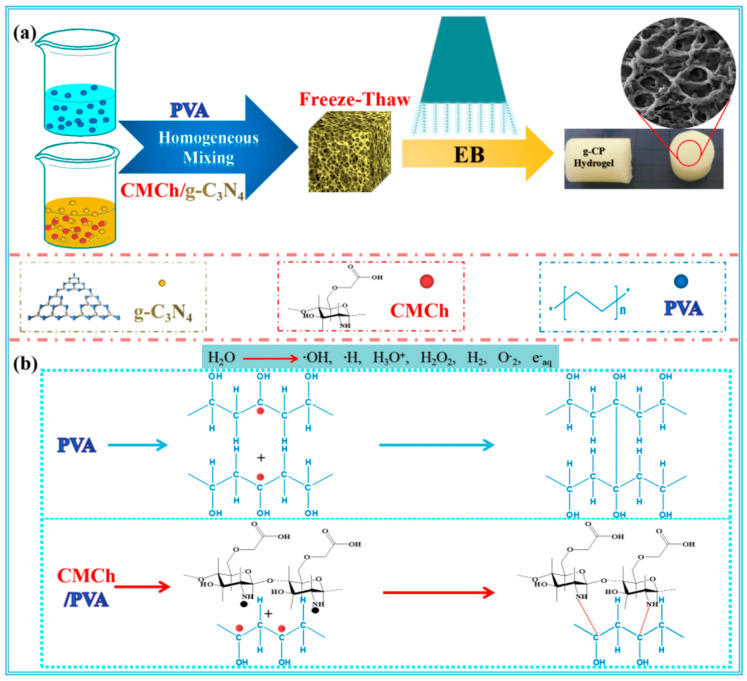
(**a**) Fabrication of g-C_3_N_4_/CHT/PVA nanocomposite hydrogel via freeze–thaw cycling followed by E. beam irradiation; (**b**) proposed synthesis mechanism of the g-C_3_N_4_/CHT/PVA nanocomposite hydrogel. Reproduced from [77] with permission from the MDPI publishers.

For instance, Chaisorn et al. synthesized a zinc oxide–AgNPs (ZA) composite and incorporated it into CMC, which was subsequently added to γ-irradiated CHT (pre-irradiated at 40 kGy) to formulate the ICZA composite hydrogel [75]. Similarly, Ali et al. developed nanocomposite hydrogels comprising CHT/poly(propenoic acid) (PPA)/ethylenediamine (EDA) in various ratios, using magnetite nanoparticles (Fe_3_O_4_ NPs) as a filler. Crosslinking was achieved via γ irradiation at a dose of 30 kGy [76]. Yang et al. prepared a graphitic carbon nitride (g-C_3_N_4_)/CHT/poly(vinyl alcohol) (PVA) hydrogel using freeze–thaw cycling followed by E. beam irradiation, as illustrated in Figure 3a. The synthesis mechanism of CM-CHT-based nanocomposite hydrogels via ionizing radiation is depicted in Figure 3b. This mechanism involves the radiolysis of water molecules induced by E. beam irradiation, generating free radicals that facilitate the interaction of PVA with g-C_3_N_4_ and CM-CHT to form a crosslinked network structure [77].

## 5. Environmental Remediation

This review highlights the use of ionizing radiation-crosslinked CHT-based hydrogels in environmental remediation. These applications include the removal of toxic heavy metals, dyes, organic contaminants, radionuclides, and humic substances. Table 1 summarizes CHT-based hydrogels fabricated through ionizing radiation for environmental remediation.

### 5.1. Removal of Toxic Heavy Metals

Wastewater treatment represents a critical application area for natural polymers. The term “wastewater” encompasses various sources, including domestic, industrial, and agricultural effluents [78,79,80]. These effluents typically contain a wide range of pollutants that pose both direct and indirect threats to human health, particularly when contaminants enter the food chain. Among these pollutants, heavy metals are among the most hazardous. These naturally occurring elements possess high atomic weights and densities at least five times greater than that of water [81,82]. Their unwanted presence in the environment stems from extensive industrial applications [83]. The five most toxic heavy metals commonly identified in wastewater are cadmium, arsenic, lead (Pb), chromium (Cr), and mercury.

To facilitate the removal of heavy metals, Wasikiewicz et al. synthesized CM-CHT and CM-chitin using E. beam irradiation for metal-ion adsorption applications [66]. The resulting hydrogels demonstrated effective adsorption of various metal cations from aqueous solutions. At pH 3.9, CM-CHT exhibited the ability to selectively separate gold (Au) cations from palladium (Pd), while CM-chitin could distinguish Pd or scandium ions from platinum ions. CM-CHT showed superior Au adsorption compared to CM-chitin, attributed to the presence of –NH_2_ groups in the CHT derivative. Based on the Langmuir model, the maximum uptake of Au cations was predicted to be 11.86 mg/g for CM-chitin and 37.59 mg/g for CM-CHT. Both materials proved advantageous for removing trace concentrations of Au ions from aqueous environments. In another study, Wang et al. successfully synthesized CM-CHT hydrogels under paste-like conditions using γ- irradiation [84]. The swelling behavior of these hydrogels in water followed Fickian diffusion and was influenced by both ionic strength and pH. Adsorption studies involving ferric ions (Fe(III)) revealed favorable uptake, facilitated by functional groups such as –OH, –NH_2_, and –COOH present in the CM-CHT matrix. Kinetic analysis indicated that the adsorption process was rapid, reaching equilibrium within approximately 20 min, and conforming to the Langmuir isotherm model. The maximum adsorption capacity for Fe(III) ions was determined to be 18.5 mg/g of hydrogel.

Similarly, Zhao et al. developed CHT/CMC hydrogels via E. beam irradiation for the adsorption of heavy metals [85]. Dissolution and swelling analyses indicated that the resulting hydrogels were insoluble in both acidic and basic environments and exhibited a relatively low swelling capacity. The incorporation of CHT enhanced hydrogel formation, increased the degree of crosslinking, and improved the adsorption performance of the CHT/CMC composites. Adsorption studies confirmed that the CHT/CMC hydrogels were effective in removing heavy metal ions, with copper (Cu) adsorption governed initially by mass transport. The adsorption isotherm was well described by the Langmuir model, suggesting monolayer adsorption. The Cu(II) adsorption mechanism involved complexation with –NH_2_ and –COOH groups on the hydrogel matrix. This study demonstrated the potential of radiation-crosslinked CHT/CMC hydrogels for the removal and recovery of heavy metal ions from aqueous systems. Additionally, Hiroki et al. prepared CM-CHT/CMC hydrogels using γ irradiation for heavy-metal adsorption applications [67]. These hydrogels exhibited high adsorption capacities for Au and Pb ions. The distribution coefficient for Au ions’ adsorption was in the range of 2.7 × 10^2^–1.8 × 10^4^, and for Pb ions it was in the range of 2.5 × 10^3^–1.1 × 10^4^. The hydrogel composition significantly influenced metal-ion uptake, with adsorption attributed to the presence of –NH_2_ groups in the CM-CHT component.

Puspitasari et al. established CHT and acrylamide (AAM)-based hydrogels via γ irradiation at room temperature for the removal of heavy metals [86]. Key parameters affecting adsorption capacity, including solution pH, contact time, initial metal ion concentration, hydrogel dosage, and the presence of competing ions, were systematically analyzed. The results demonstrated that the CHT-co-PAAM hydrogels exhibited excellent adsorption capabilities, with ion uptake significantly influenced by all of the investigated parameters. The order of heavy-metal ion uptake was Zn^2+^ (296.68 mg/g) > Cr^6+^ (242.77 mg/g) > Pb^2+^ (187.64 mg/g) > Cu^2+^ (151.93 mg/g) > Co^2+^ (127.86 mg/g) > Ni^2+^ (59.29 mg/g). The adsorption data conformed well to both the Langmuir and the Freundlich isotherm models.

In another study, Elbarbary et al. prepared 2-hydroxyethyl methacrylate (HEMA) and CHT interpenetrating polymer network (IPN) hydrogels using γ-irradiation and chemical modification through phosphorylation reaction [87]. The developed IPN was employed to adsorb Cu(II), zinc (Zn(II)) ions, and calcium (Ca(II)) from the respective solutions. IPN showed a highly porous structure with white fibrils separated by interconnected pores. The phosphorylation-modified hydrogels maintained high removal efficiency (86%) over five adsorption cycles. The maximum adsorption capacities for Ca(II), Zn(II), and Cu(II) ions using 0.01 g of Phos-(CHT/PHEMA) were 48.7, 57.6, and 66.3 mg/g, respectively. The adsorption behavior followed the Langmuir and the Freundlich isotherm models, while the kinetics adhered to the pseudo-second-order model. The findings confirmed that chemical modification significantly improved the adsorption capacity, highlighting the possibility of use for the effective removal of metal ions from aqueous media.

**Figure 4:**
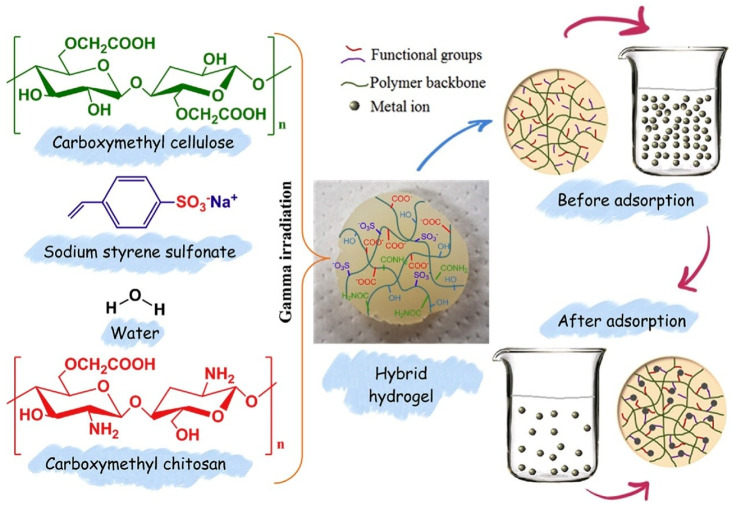
Fabrication of CM-CHT/CMC/SSS-based hybrid hydrogels via γ irradiation and depiction of Ag^+^ ion interaction. Reproduced from [88] with permission from Elsevier publishers.

Tran et al. developed CM-CHT/CMC/sodium sulfonate styrene (SSS)-based hybrid hydrogels via γ irradiation [88]. These hydrogels were employed as sorbents for Ag^+^ ions under both competitive and non-competitive conditions, as illustrated in Figure 4. Initially, optimal adsorption conditions, such as (CM-CHT/CMC):SSS ratio, pH, and contact time, were found to be 4:2, pH 5, and 10 h, respectively. The experimental data were analyzed using equilibrium isotherm models (Freundlich, Temkin, and Langmuir) to determine the maximum Ag^+^ uptake. The adsorption data fitted well with the Langmuir isotherm model and estimated maximum adsorption of 451.74 × 10^−3^ mg/g due to monolayer adsorption. Adsorption kinetics were evaluated using both pseudo-first-order and pseudo-second-order models to elucidate the underlying mechanisms. It was found that the adsorption process follows pseudo-second-order models. The reusability results revealed that Ag+ adsorbate can be removed by 0.01 M HNO_3_ (desorbing agent), the adsorption percentage was not changed after four cycles, and the desorption values were over 82%. The thermal effects on adsorption were assessed to determine the thermodynamic nature of the process, which was found to be endothermic and spontaneous. These results highlight the potential application of the developed hydrogels in wastewater treatment processes involving silver ions.

**Figure 5:**
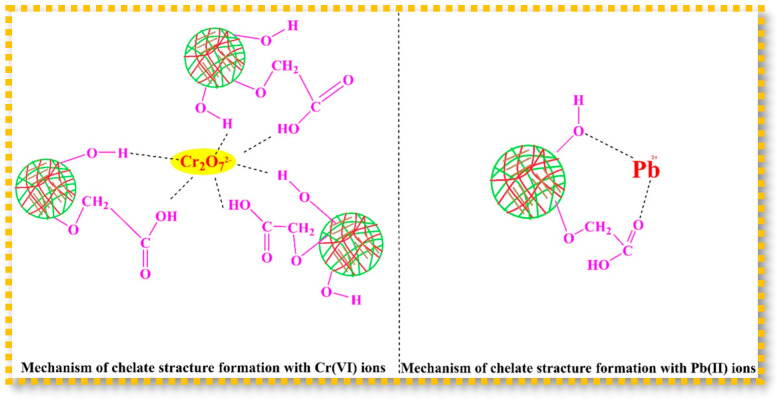
Proposed mechanism for uptake process of Cr(VI) and Pb(II) ions. Reproduced from [89] with permission from Springer Nature Link publishers.

Similarly, El-Sayed Abdel-Raouf et al. developed eco-friendly CM-CHT/acrylic acid (AA)/nanoclay nanocomposite hydrogels via γ-irradiation for the removal of highly toxic metals [89]. Under optimal conditions, the hydrogels achieved maximum adsorption capacities of 205 mg/g for Cr(VI) at pH 9 and 125 mg/g for Pb(II) at pH 8 within 90 min. The proposed adsorption mechanisms for Cr(VI) and Pb(II) are illustrated in Figure 5. Kinetic analysis indicated that the adsorption process followed a pseudo-first-order model. Furthermore, the nanocomposite hydrogels retained satisfactory metal uptake capacity over three successive adsorption–desorption cycles. The adsorption behavior was consistent with the Langmuir isotherm model, and the mechanism was attributed to electrostatic interactions and chelation between the metal ions and reactive functional groups on the sorbent matrix. Thermodynamic analysis confirmed that the adsorption process was spontaneous.

### 5.2. Removal of Dyes

**Figure 6:**
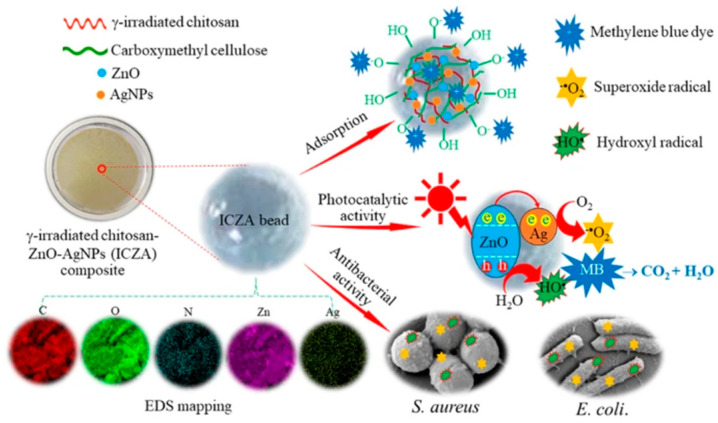
Schematic illustration of ICZA composite hydrogel formation and its proposed mechanisms for adsorption, photocatalytic activity, and antibacterial performance. Reproduced from [75] with permission from Springer Nature Link publishers.

Dyes are complex compounds that naturally adsorb onto the surface of substrates, imparting color, which renders them valuable for various industrial applications, including paper, pulp, textiles, and cosmetics [90,91]. They are broadly classified into two categories—natural and synthetic—encompassing both anionic and cationic dyes [92]. Global dye consumption is estimated at approximately 10,000 tons per annum, a figure that continues to rise with an increasing population and industrial demand [93]. The discharge of untreated dye-containing wastewater into aquatic systems poses serious environmental and health risks, including toxicity to aquatic organisms and adverse human effects such as tumors and skin allergies [76]. In response, nanocomposite adsorbents have gained widespread application for dye removal due to their advantageous properties, including uniform particle size, chemical stability, and biocompatibility [94]. Chaisorn et al. developed an ICZA composite hydrogel through a simple fabrication method [75]. This composite was evaluated for its antibacterial activity and its ability to adsorb and photodegrade organic dyes. The ICZA hydrogel exhibited a high adsorption capacity for methylene blue (MB), with a maximum uptake of 92.59 mg/g, and its adsorption behavior conformed to the Langmuir isotherm model. Kinetic studies revealed that MB adsorption conformed to a pseudo-first-order model. The efficiency of hydrogels was minimally reduced after five cycles. Furthermore, it demonstrated superior photocatalytic degradation performance under UV light compared to other catalysts, with an apparent rate constant of 3.08 × 10^−2^. The composite also showed strong antibacterial activity against *Staphylococcus aureus* (*S. aureus*), with minimum inhibitory and bactericidal concentrations of 12.5 g/mL and 50 g/mL, respectively, under both dark and light conditions. The underlying mechanisms of the ICZA composite’s adsorption, photocatalytic, and antibacterial activities are illustrated in Figure 6.

Likewise, Ali et al. developed CHT/PPA/EDA/Fe_3_O_4_ NPs-based nanocomposite hydrogels using a γ radiation technique for wastewater contamination remediation applications [76]. The resulting hydrogels exhibited both superparamagnetic and superabsorbent properties. Adsorption kinetics for the Lerui acid brilliant blue (LABB) and Astrazon blue (AB) dyes followed a pseudo-second-order model, while thermodynamic analysis revealed endothermic adsorption behavior. The adsorption data were best described by the Langmuir isotherm model. The nanocomposite hydrogel demonstrated high adsorption capacities, effectively removing toxic and hazardous dyes, with uptake values of 51.9 mg/g for the acidic dye LABB and 193.21 mg/g for the basic dye AB. The results of regeneration studies reveal that the desorption percentage was high after three cycles, with a small decrease in the adsorption and desorption capacities. These results underscore the hydrogel’s substantial potential for removing harmful dyes from contaminated wastewater. In another study, Yang et al. developed a dark/light dual-mode photoresponsive g-C_3_N_4_/CHT/PVA sterilized nanocomposite hydrogel using freeze–thaw cycling followed by E. beam radiation [77]. Photocatalytic testing showed that the hydrogel degraded rhodamine B dye by 65.92% within 60 min, and the adsorption kinetics studies obeyed the quasi-second order fitting model. Additionally, it exhibited strong antimicrobial activity against *S. aureus* and *Escherichia coli* within 4 h. The hydrogel could be molded into various shapes—including cylinders, bars, and cubes—and displayed excellent biocompatibility and mechanical strength, with compressive modulus and tensile strength measured at 1.61 MPa and 0.093 MPa, respectively. Biocompatibility was confirmed using Hoechst 33342/PI double staining and the Cell Counting Kit-8 assay. This multifunctional hydrogel shows strong potential for applications in wound dressings and environmental wastewater treatment.

In addition, Helal et al. synthesized PVA/CMC/CHT-based hydrogels using γ irradiation at varying doses and investigated their biodegradability and ability to remove ionic dyes [95]. These hydrogels were employed to extract MB and direct blue 1 (DB1) from aqueous solutions. The adsorption behavior of both dyes followed the Langmuir isotherm model. The maximum adsorption capacity for MB was 8.09 mg/g and for DB1 it was 17.23 mg/g. Kinetic studies revealed that MB adsorption conformed to a pseudo-first-order model, whereas DB1 followed a pseudo-second-order kinetic model. The efficiency of hydrogels was minimally reduced after five cycles. Biodegradation analysis showed that the pristine hydrogels exhibited faster soil degradability than those loaded with MB and DB1. Regarding their water-holding capacity and water retention in sandy soils, the hydrogels synthesized at 20 kGy demonstrated the highest performance, while those prepared at 2.5 kGy exhibited the lowest.

### 5.3. Extraction of Organic Contaminants

Environmental pollution caused by the improper disposal of organic solvents and oil spills poses a significant threat to ecosystems and global water resources [96,97]. Even in trace concentrations, these pollutants can exert long-term and severe effects by disrupting the delicate balance of aquatic ecosystems and jeopardizing the health of communities reliant on these water sources [80,98,99]. To address this issue, Ghobashy et al. developed multi-walled CNTs-based aerogels as efficient adsorbents for removing various organic solvents and pumping oil from water. In their study, CHT/PAAM aerogels were synthesized via γ irradiation in the presence of sodium silicate, incorporating different concentrations of CNTs to enhance oil adsorption efficiency [100]. γ-irradiation facilitated the formation of porous and lightweight structures, while the chemical reagent method posed a risk of altering CNTs’ morphology and compromising porosity. Physicochemical analyses confirmed the successful incorporation of CNTs, uniform dispersion, hydrophobicity, enhanced compressive strength, and increased surface area. The CNTs-incorporated aerogels achieved methanol and oil removal efficiencies of 80.68% and 93.15%, respectively, within 60 min. The efficiency of hydrogels was minimally reduced after five cycles. Kinetic studies indicated a chemisorption mechanism, underscoring the role of CNTs in facilitating improved chemical interactions, and followed the pseudo-second-order model.

### 5.4. Removal of Radionuclides

The expanding use of radioactive isotopes in medical imaging and radiation therapy, along with the substantial volume of hazardous nuclear waste generated, raises significant environmental concerns. Common techniques for treating radioactive waste include chemical precipitation, filtration, ion exchange, adsorption, and evaporation. Among these, adsorption is considered one of the most effective methods for removing radionuclides, owing to the selectivity, low cost, accessibility, and high efficiency of adsorbent materials in the remediation of liquid radioactive waste [101]. In this context, Zhuang et al. synthesized CHT-g-maleic acid hydrogels via γ-radiation-induced grafting for the removal of radioactive cobalt (^60^Co) ions [102]. The study evaluated the effects of monomer concentration and irradiation dose on the grafting percentage, as well as the isotherms and adsorption kinetics. The experimental data for ^60^Co adsorption conformed well to the Temkin isotherm model (R^2^ = 0.96) and the pseudo-second-order kinetic model (R^2^ = 0.99). The grafting process enhanced the equilibrium adsorption capacity of ^60^Co ions from 2.00 mg/g to 2.78 mg/g.

**Figure 7:**
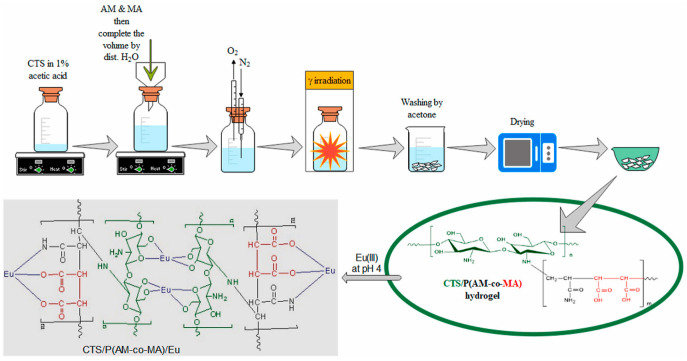
Schematic representation of CHT/P(AAM-co-MA) copolymer hydrogel synthesis via γ irradiation and the proposed bonding mechanism of Eu(III) with the CHT/P(AAM-co-MA) hydrogel. Reproduced from [103] with permission from Elsevier.

In another study, Abdelmonem et al. developed a CHT/poly(acrylamide-co-maleic acid) [P(AAM-co-MA)] copolymer hydrogel using γ irradiation, as illustrated in Figure 7 [103]. The synthesized hydrogel rapidly adsorbed europium ^152+154^Eu(III) ions from aqueous solution, reaching equilibrium within 24 h at a pH of approximately 4. The adsorption process closely followed the Langmuir isotherm, exhibiting a maximum capacity of 144.96 mg/g. Kinetic studies showed that the adsorption behavior was best described by the pseudo-second-order model. Thermodynamic analysis revealed that the process was exothermic, spontaneous, and more efficient at lower temperatures. Desorption of ^152+154^Eu(III) ions was achieved with efficiencies of 88.63% using aluminum chloride and 97.09% using 0.1 M of hydrochloric acid. These findings suggest that the CHT/P(AAM-co-MA) hydrogel is a promising candidate for future applications in the adsorption of trivalent lanthanide ions.

### 5.5. Removal of Humic Substances

Humic substances can adversely affect water quality not only by imparting undesirable color but also by forming complexes with metal ions—potentially raising their concentrations above acceptable limits—and by reacting with chlorine to produce trihalomethanes [104]. Therefore, minimizing the presence of humic substances in drinking and industrial water is essential. In this context, Zhao et al. fabricated irradiation crosslinked CM-CHT hydrogels using E. beam irradiation without the use of additives [105]. Dissolution tests demonstrated that the resulting hydrogels were insoluble in acidic, basic, and certain organic solvents. The crosslinked hydrogels exhibited a positive zeta potential at pH values below six, primarily due to the protonation of –NH_2_ groups. Adsorption studies showed that the irradiation crosslinked CM-CHT hydrogels were effective adsorbents for humic acid removal under acidic conditions, with optimal performance at pH 3.5. The protonated –NH_2_ groups played a critical role in forming surface complexes with humic acid, thereby facilitating the adsorption process. Isotherm analysis confirmed that the experimental data conformed well to the Langmuir model. These findings demonstrate the potential of irradiation crosslinked CM-CHT hydrogels for the concentration and separation of humic acid in water treatment applications.

**Table 1:** Overview of CHT-based hydrogels synthesized using ionizing radiation for environmental remediation.

Materials	Ionizing Radiation (Parameters)	Adsorption Kinetics, Isotherms, and Examined Number of Cycles	Application (Potential for Adsorbate Removal)	References
Poly(vinyl alcohol); Sodium carboxymethyl cellulose; Chitosan	γ radiation radiation doses: 2.5, 5, 10, and 20 kGy; (dose rate: 2.5 kGy/h)	MB adsorption: pseudo-first-order, DB1 adsorption: pseudo-second-order kinetic model; Langmuir model; 5 cycles	Removal of ionic dyes and their biodegradation (8.09 mg/g of methylene blue and 17.23 mg/g of direct blue)	[95]
Poly(acrylamide); Chitosan; Multi-walled carbon nanotubes	γ radiation radiation dose: 30 kGy; (dose rate: 0.6 kGy/h)	Pseudo-second-order model; 5 cycles	Potential for effectively removing oil and organic solvent contaminants from water (93.15% of oil and 80.68% of methanol)	[100]
Chitosan; Zinc oxide; Silver nanoparticles	γ radiation radiation dose: 40 kGy	Pseudo-first-order kinetic model; Langmuir isotherm model; 5 cycles	Organic dye removal (92.59 mg/g of methylene blue)	[75]
Graphitic carbon nitride; Chitosan; Poly(vinyl alcohol)	E. beam radiation dose: 30 kGy; (dose rate: 5 kGy/pass)	Quasi-second-order fitting	Potential for environmental wastewater treatment (65.92% of rhodamine B)	[77]
Chitosan; Maleic acid	γ radiation radiation dose: 1–5 kGy; (dose rate: 31.98 Gy/min)	Pseudo-second-order kinetic model; Temkin isotherm model	Potential for adsorption of radioisotopes (2.78 mg/g of cobalt-60)	[102]
Chitosan; Poly(acrylamide-co-maleic acid)	γ radiation radiation dose: 20 kGy	Pseudo-second-order model Langmuir isotherm	Potential for adsorption of trivalent lanthanide ions (144.96 mg/g of ^152+154^Eu (III))	[103]
N,O carboxymethyl Chitosan; Nanoclay; Acrylic acid	γ radiation radiation dose: 25 kGy	Pseudo-first-order model; Langmuir isotherm model; 3 cycles	Removal of Cr(VI) and Pb(II) from aqueous media (205 mg/g of Cr(VI) and 125 mg/g of Pb(II))	[89]
Chitosan; Poly(propenoic acid); Ethylenediamine; Magnetite	γ radiation radiation dose: 30 kGy; (dose rate: 0.309 Gy/s)	Pseudo-second-order model; Langmuir isotherm model; 3 cycles	Potential for wastewater contamination remediation applications (193.21 mg/g of Astrazon blue and 51.9 mg/g of Lerui Acid Brilliant Blue dye)	[76]
Carboxymethyl chitosan; Carboxymethyl cellulose; Sodium sulfonate styrene	γ radiation radiation dose: 60 kGy	Pseudo-second-order models; Langmuir isotherm model; 4 cycles	Potential for wastewater treatment processes containing silver metal (451.74 × 10^−3^ mg/g of silver)	[88]
Chitosan; 2-Hydroxyethyl methacrylate	γ radiation radiation dose: 10, 20, and 30 kGy; (dose rate: 1.95 kGy/h)	Pseudo-second-order model; Langmuir and the Freundlich isotherm models; 5 cycles	Metal ions removal 66.3 mg/g of Cu(II), 48.7 mg/g of Ca(II), and 57.6 mg/g Zn (II)	[87]
Chitosan; Acrylamide	γ radiation radiation dose: 10–40 kGy	Langmuir and the Freundlich isotherm models	Metal ions removal (296.68 mg/g of Zn^2+^, 242.77 mg/g of Cr^6+^, 187.64 mg/g of Pb^2+^, 151.93 mg/g of Cu^2+^, 127.86 mg/g of Co^2+^ and 59.29 mg/g of Ni^2+^)	[86]
Carboxymethyl cellulose; Carboxymethyl chitosan	γ radiation radiation dose: 10–200 kGy; (dose rate: 10 kGy/h)	–	Metal ions removal Distribution coefficient (2.5 × 10^3^–1.1 × 10^4^ of Pb and 2.7 × 10^2^–1.8 × 10^4^ of Au)	[67]
N,O-carboxymethyl chitosan	γ radiation radiation dose: 0–100 kGy	Langmuir isotherm model	Fe(III) ion adsorption (18.5 mg/g of Fe(III))	[84]
Carboxymethyl chitosan	E. beam radiation dose: 50–200 kGy; (dose rate: 1 kGy/pass)	Langmuir model	Potential in water treatment for the removal of humic acid and electrically charged or other polarized species (57.14 mg/g of humic acid)	[105]
Chitosan; CM-cellulose	E. beam radiation dose: 10–100 kGy; (dose rate: 5 kGy/pass)	Langmuir model	Potential for applications in water treatment for the removal of heavy metal ions (169.49 mg/g of Cu(II))	[85]
Carboxymethyl chitin; Carboxymethyl chitosan	E. beam radiation dose: 10 up to 200 kGy	Langmuir equation	Adsorption of metal ions (37.59 mg/g of Au for CM-CHT, 11.86 mg/g of Au for CM-chitin)	[66]

## 6. Conclusion, Challenges, and Future Perspectives

This review focuses on ionizing radiation-crosslinked CHT-based adsorbents useful across several contaminant removal areas, showcasing promising results and important innovations in environmental remediation. The use of ionizing radiation in hydrogel fabrication offers several advantages, including reduced reliance on toxic chemicals and precise control over hydrogel properties. However, limitations persist, such as the high cost of radiation equipment and the requirement for specialized facilities. Also, investigations on optimizing radiation-dosage parameters and polymeric concentration are required.

Despite notable progress in environmental remediation using ionizing radiation-processed CHT-based hydrogels, challenges remain, and opportunities for further optimization exist. Focus should be given to improving the mechanical strength, stability, and adsorption efficiency and selectivity of these hydrogels. The development of multifunctional CHT-based hydrogels through the incorporation of structural or functional variants could further enhance their performance. After the adsorption tendencies of an adsorbent, reusability is of prime importance. In the literature, after several usage cycles, only the adsorption data is measured. In this regard, the impact of regeneration cycles on the morphology and mechanical strength of the adsorbents should be monitored to evaluate the reusability of the adsorbents. The majority of studies carried out adsorption experiments using one or two types of heavy metal, which makes it difficult to evaluate the selectivity of the adsorbent. The utilization of CHT-based adsorbents can be expanded to a wide variety of pollutants. New applications could be explored and employed in real-world wastewater treatment to investigate their efficacy. Furthermore, a comprehensive assessment of market feasibility and economic viability is recommended. Continued research and development efforts are essential to fully unlock the full potential of CHT-based hydrogels and overcome existing technological barriers.

## Data Availability

No new data were created or analyzed in this study.
